# Relative abundance of *Mycobacterium bovis* molecular types in cattle: a simulation study of potential epidemiological drivers

**DOI:** 10.1186/s12917-017-1190-5

**Published:** 2017-08-22

**Authors:** Hannah Trewby, David M. Wright, Robin A. Skuce, Carl McCormick, Thomas R. Mallon, Eleanor L. Presho, Rowland R. Kao, Daniel T. Haydon, Roman Biek

**Affiliations:** 1 Boyd Orr Centre for Population and Ecosystem Health, Institute of Biodiversity and Animal Health, University of Glasgow, Glasgow, UK; 2 School of Medicine, Dentistry, and Biomedical Sciences, Queen’s University Belfast, Belfast, UK; 3 Veterinary Sciences Division, Agri-Food and Biosciences Institute, Stormont, Belfast, UK; 4 School of Biological Sciences, Queen’s University Belfast, Belfast, UK

**Keywords:** Bovine tuberculosis, Multiple-locus variable number tandem repeats, Neutral theory of biodiversity, Species abundance distributions, Population genetics

## Abstract

**Background:**

The patterns of relative species abundance are commonly studied in ecology and epidemiology to provide insights into underlying dynamical processes. Molecular types (MVLA-types) of *Mycobacterium bovis,* the causal agent of bovine tuberculosis, are now routinely recorded in culture-confirmed bovine tuberculosis cases in Northern Ireland. In this study, we use ecological approaches and simulation modelling to investigate the distribution of relative abundances of MVLA-types and its potential drivers. We explore four biologically plausible hypotheses regarding the processes driving molecular type relative abundances: sampling and speciation; structuring of the pathogen population; historical changes in population size; and transmission heterogeneity (superspreading).

**Results:**

Northern Irish herd-level MVLA-type surveillance shows a right-skewed distribution of MVLA-types, with a small number of types present at very high frequencies and the majority of types very rare. We demonstrate that this skew is too extreme to be accounted for by simple neutral ecological processes. Simulation results indicate that the process of MVLA-type speciation and the manner in which the MVLA-typing loci were chosen in Northern Ireland cannot account for the observed skew. Similarly, we find that pathogen population structure, assuming for example a reservoir of infection in a separate host, would drive the relative abundance distribution in the opposite direction to that observed, generating more even abundances of molecular types. However, we find that historical increases in bovine tuberculosis prevalence and/or transmission heterogeneity (superspreading) are both capable of generating the skewed MVLA-type distribution, consistent with findings of previous work examining the distribution of molecular types in human tuberculosis.

**Conclusion:**

Although the distribution of MVLA-type abundances does not fit classical neutral predictions, our simulations show that increases in pathogen population size and/or superspreading are consistent with the pattern observed, even in the absence of selective pressures acting on the system.

**Electronic supplementary material:**

The online version of this article (doi:10.1186/s12917-017-1190-5) contains supplementary material, which is available to authorized users.

## Background

Bovine tuberculosis (bTB) is one of the most important diseases facing the livestock industry in Britain and Ireland. Control is complicated by infection in a wildlife reservoir, the Eurasian badger [1–3], and there has been much debate regarding the relative roles of cattle and badgers in driving pathogen population dynamics. Analysis of the relative abundance distributions (RADs) of pathogen molecular types could provide insight into the mechanisms underlying bTB spread. These techniques originated in the ecological literature to explain patterns of species diversity and are presently under-used in epidemiology, despite the wealth of data on the relative abundances of pathogen molecular types collected for diseases of both human and animal importance.

Analysis of abundance distributions should be applied with caution however, as RADs in isolation do not necessarily provide sufficient information to identify or distinguish between the specific ecological or epidemiological events that shape them [4–6]. In fact, simple neutral processes, such as those outlined in Hubbell’s neutral theory of biodiversity and biogeography (hereafter referred to as NTB [7]), have shown a surprising ability to predict the RADs recorded in a wide variety of ecological datasets [8].

NTB is a theory of “ecological drift” and, in ecology, is traditionally set against the non-neutral hypotheses of niche theory and selective pressures. It is based around the idea of per-capita equivalence: all individuals, irrespective of species, have an equal chance of birth, death, reproduction, and immigration and/or speciation (with the analytical solutions put forward by Hubbell also relying on an assumption of constant population size) [7]. Further developments have extended the theory to consider the explicit spatial structure of the population through spatially limited dispersal of individuals [7, 8]. Although it is widely accepted that real ecosystems are not truly neutral and species are not identical, the success of NTB demonstrates that simple processes are often sufficient to drive patterns of diversity observed in natural systems.

RADs have also been considered in some epidemiological contexts, for example to examine the relative abundance of different molecular types of tuberculosis-causing mycobacteria in human host populations. As with ecological datasets, the observed distributions tend to be right-skewed, with a small number of molecular types present at high frequency and the majority of strains being rare. Luciani et al. showed that the RADs seen in several human tuberculosis datasets did not fit the predictions generated by NTB, and demonstrated that an increasing bacterial population size could account for the distribution of at least one of these datasets [9]. Ypma et al. also examined the RAD of different molecular types of human tuberculosis, this time in the Netherlands. They argued that an increasing bacterial population was unlikely in this system, but demonstrated that variation in the numbers of offspring per infection (superpreading) could explain the skewed distribution observed [10]. In a rare example from veterinary epidemiology, Smith et al. suggested that “clonal expansion” of certain bacterial lineages, for example due to natural selection or the expansion of lineages into new host species or geographical areas, accounted for the RAD of different molecular types of *Mycobacterium bovis*, the causal agent of bTB, recorded in cattle in Great Britain (GB) [11].

In this study we extend these approaches, exploring four biologically plausible hypotheses to examine the processes driving molecular type relative abundances of *M. bovis* in the Northern Ireland (NI) cattle population. BTB is endemic in cattle in Northern Ireland [12], and intensive molecular typing of *M. bovis* cultures from culture-confirmed bTB breakdowns has been carried out since 2003 [13]. Similar to the molecular types of *M. bovis* in GB [11], molecular types in NI have shown a marked right-skewed RAD [14], however the possible epidemiological processes underlying this skew have not been examined. In common with *M. bovis* in other parts of the UK and Ireland [15–18], infections in NI also give the visual impression of clustering geographically by molecular type [13].

Molecular typing of *M. bovis* in NI is conducted through a combination of spoligotyping, based on the presence of multiple spacer oligonucleotides within the Direct Repeat region of the genome [19], and Multiple-Locus Variable Number Tandem Repeat Analysis (MVLA)-typing [20, 21], which measures the number of repeats at various MVLA loci present in the mycobacterial genome. MVLA-typing gives a higher level of discrimination than spoligotyping, although both methods are prone to a degree of homoplasy, i.e. the occurrence of the same MVLA-type in unrelated lineages [22, 23]. In NI, the specific MVLA-loci used in MVLA-typing have been chosen to optimise the discriminatory power of the technique in this population [14], and MVLA-types are used in conjunction with spoligotyping. Throughout this paper the molecular type of an isolate, as determined by the combination of spoligotype and MVLA-type results, is referred to as the “MVLA-type”.

*M. bovis* as a species shows very little genetic diversity, especially in Britain and Ireland [15]. Studies in NI have identified no significant differences between the molecular types of *M. bovis* in either the size of herd outbreaks or in the response to the bTB skin test, although there are indications that the number and anatomical distribution of bTB lesions may differ by molecular type [24, 25]. In a low diversity population such as that in NI, selective pressures are less likely to play a prominent role in shaping the diversity of the *M. bovis* population. However, historical increases in bTB incidence over recent decades [26] may have affected the relative abundances of the different molecular types. Furthermore, superspreading at the herd level has been indicated to play a role in the spread of the disease in Great Britain [27], and this may also impact the RAD observed here.

In this study we investigate the processes that potentially underlie the RAD of molecular types of *M. bovis* isolated from cattle in NI. We first evaluate the null hypothesis that the observed RAD of molecular types in NI can be accounted for by neutral processes alone, as put forward in NTB, by comparing the observed distribution of molecular types to that predicted under NTB. We go on to use simulation models to examine four alternative hypotheses regarding mechanisms involved in shaping the distribution of MVLA-types in NI: 1) the process of MVLA-type speciation and/or the manner in which MVLA-loci were chosen; 2) the existence of an unsampled pool of infection in a separate but linked population (for example a wildlife reservoir); 3) the recent expansion of the *M. bovis* population [9]; and 4) superspreading, i.e. variability in the number of onward infections transmitted from an infected herd [10].

## Methods

All analyses and simulations were conducted in R v3.3.1 [28].

### Data on molecular types of *M. bovis* in NI

In NI, all cattle over the age of 6 weeks are routinely tested for bTB on an annual basis, and bTB surveillance is additionally conducted routinely at slaughter. A “breakdown” of bTB in a cattle herd is defined as the period of time beginning with the first detection of bTB in an animal from the herd, and ending when the entire herd has passed two consecutive bTB tests, the first completed at least 60 days after removal/isolation of the infected animal and the second at least 120 days after removal/isolation of the infected animal (if the breakdown was not confirmed, this may be reduced to a single bTB test completed at least 60 days after removal/isolation of the infected animal), plus completion of cleansing and disinfection of the premises as specified by notice. Since 2003, all NI bTB breakdowns from which *M. bovis* was successfully cultured have had at least one isolate typed using both spoligotyping and MVLA-typing using standard protocols [14]. In NI, the specific loci used for MVLA-typing are MV2163B/QUB11B, MV4052/QUB26A, MV2461/ETRB, MV2165/ETRA, MV2163/QUB11A and MV323/QUB3232, although since 2006 an extra MVLA locus, MV1895/QUB1895, has been added to the NI panel in order to split one of the common types into two geographically distinct types [13]. To ensure consistency across the sampled timeframe, in this study we consider MVLA-types as defined using the original seven-locus MVLA panel plus spoligotype.

Details of herd breakdowns, restricted to those breakdowns beginning in the years 2003–2010 inclusive, were made available from the APHIS database [29]. Each molecular typing record was assigned to a breakdown based on date and herd identifier, and the dataset was downsampled to include only the first typing record for each breakdown, as multiple isolates per breakdown were only routinely typed after 2008. A total of 10,049 out of 17,484 breakdowns (57.5%) for this period were linked to at least one molecular typing record (Table 1), and 18,699 out of 20,322 molecular typing records (92%) were successfully assigned to a breakdown (2656 typed breakdowns had more than one molecular typing result recorded). Sixteen unique spoligotypes (Additional file 1) and 183 unique MVLA-types (Additional file 2) were recorded over the study period.

**Table 1:** Numbers of *M. bovis* spoligotypes, MVLA-types, and typed breakdowns recorded by year in NI

Year	No. unique spoligotypes	No. unique MVLA-types	No. typed breakdowns	Total no. of breakdowns
2003	12	57	1347	2902
2004	13	61	1621	2954
2005	12	61	1432	2556
2006	9	72	1285	2033
2007	10	72	1235	1862
2008	11	72	1115	1789
2009	10	72	1080	1771
2010	9	72	934	1617
Total (unique) 2003–2010	16	185	10,049	17,484

### Null hypothesis - neutral ecological drift

The R package untb [30] was used to test whether the observed RAD of *M. bovis* molecular types conformed to predictions generated by NTB. This package applies the theory put forward by Hubbell [7] to generate stochastic realisations of the predicted RAD for a set group of species. This is achieved by first calculating the fundamental biodiversity number *θ*, which is estimated from the number of species present in the community *S* using statistical inference based on Ewens’ sampling formula [30, 31]. Theta is a composite parameter discussed extensively by Hubbell [7], and is related to the total number of individuals present in the community (*J*_*M*_) and the speciation rate (*ν*) by $\theta ={J}_M\frac{\nu }{1-\nu }$ [8]. When *θ* is combined with *J*_*M*_, it allows stochastic generation of predicted RADs expected for the community under neutral theory (in the absence of dispersal limitation), using the algorithm specified by Hubbell ([7], p289). Predicted RADs are calculated without direct reference to the relative abundances observed in the community under study, therefore comparison between these predictions and the observed RADs provide a means to test whether the observed abundances deviate from the distribution expected under NTB [8].

For the purposes of this analysis, different molecular types of *M. bovis* were treated as different species, and the number of herd breakdowns of each molecular type in NI was taken to represent the number of individuals per species. Molecular types were defined based on either spoligotyping alone (spoligotypes), or combined spoligotyping and MVLA-typing (MVLA-types). Using the untb package, the fundamental biodiversity number *θ* was first estimated from the observed RAD, and taking this value of *θ* and the total number of typed herd breakdowns (*n* = 10,049), 1000 stochastic realisations of the RADs predicted under NTB were generated. The 95th percentile interval of these distributions was then plotted to compare the observed RAD with NTB predictions for the community.

The above steps were carried out for the RADs of: i) all recorded *M. bovis* spoligotypes across the entire study period (2003–2010); ii) all recorded MVLA-types across the entire study period; and iii) MVLA-types subdivided by year of observation (the latter to identify whether there were differences between RADs over the course of the study period and to what extent this subdivision affected the fit to neutral predictions).

### Alternative hypotheses - basic simulation model structure

To test alternative hypotheses regarding the processes involved in shaping the distribution of MVLA-types in NI, four stochastic simulation models were constructed. Specific details of each model are given in the sections below, but we describe the basic model structure here as it is comparable across all subsequent models. Within the model, a single ‘individual’ represents a single (MVLA-typed) herd breakdown of bTB, and species equates to MVLA-type (defined by the combination of spoligotype and MVLA-type). A speciation event represents a mutation in MVLA-type, and ‘birth’ and ‘death’ events are the start and end of herd breakdowns respectively, with each new breakdown infected from a “parent” individual. The population size in the model is equivalent to the number of (MVLA-typed) bTB breakdowns on herds at any one time, and the birth rate is equivalent to the herd-level incidence rate.

The total population size at time *t* is given by: ${J}_{M,t}=\sum_{i=1}^{S_t}{n}_{i,t}$, where *S*_*t*_ is the total number of species present at time *t*, *n*_*i,t*_ is the number of individuals in the *i*th species at time *t*, and *i =* 1…*S*_*t*_. Simulations were started with individuals in the population at one of two extremes: either all individuals started as the same species, with *S*_0_ = 1 and *n*_*i,*0_ = *J*_*M*,0_; or every individual started as a different species, with *S*_0_ = *J*_*M*,0_ and *n*_*i,*0_ = 1. Comparing results from both starting conditions allowed us to check the model for convergence.

**Fig. 1:**
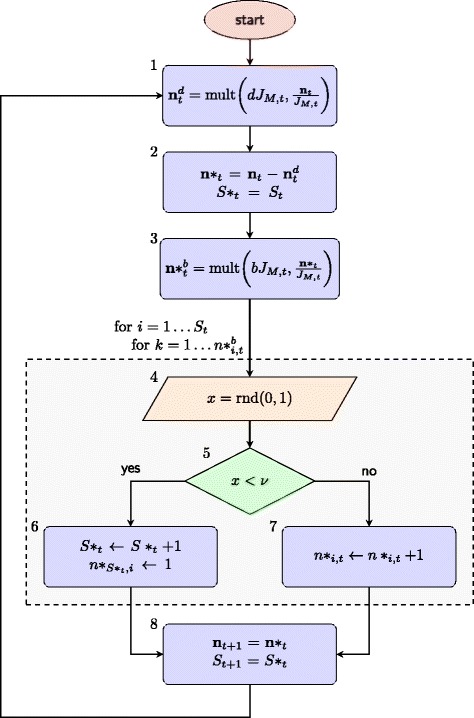
Flowchart to show the steps carried out for the basic model, which simulates the evolution of a community of individuals of different species undergoing neutral ecological drift. The number of individuals in each species at time *t i*s represented by the vector ***n***_*t*_, where the element *n*_*i* , *t*_ is the number of individuals of species *i* at time *t*, and *i* = 1 , … , *S*_*t*_. ${\boldsymbol{n}}_t^d$ is the vector of individuals that are removed from each of the species in the model at time *t* (boxes 1 and 2). The vector containing the number of individuals in each species at intermediate timepoints is indicated by ***n*** ^∗^, and the number of species in the population at intermediate timepoints is *S* ^∗^_*t*_. ${{\boldsymbol{n}}^{\ast}}_t^b$ (box 3) is the vector of individuals in each species chosen to reproduce at time *t. ν* is the probability of speciation occurring per birth event (box 5). Total population size at time *t* (boxes 1 and 3) is given by ${J}_{M,t}=\sum_{i=1}^{S_t}{n}_{i,t}$. Function mult(*x,* ***y***) (boxes 1 and 3) generates a multinomially distributed random number vector of size *x*, where ***y*** is a vector giving the probability of drawing the individuals from each class. Function rnd.(0,1) (box 5) generates a random variable distributed evenly between 0 and 1. The dashed rectangle indicates steps conducted while looping through each birth *k* in each species *i*

An overview of the steps involved in one timestep of the basic simulation model is given in Fig. 1. First, a vector, ${\boldsymbol{n}}_t^d$, representing the number of individuals in each species that “die” in the current timestep, is identified (box 1, Fig. 1) and removed from the population (box 2, Fig. 1). The number of individuals to be removed is given by the death rate *d* multiplied by the total population size *J*_*M,t*_ (box 1). A vector representing the number of new births in each species at this timestep, ${\boldsymbol{n}}_t^b$, is then identified, with the total number of individuals to be born given by the birth rate *b* multiplied by total population size *J*_*M,t*_. The number of offspring per species in the basic model is proportional to the frequency of that species in the population (box 3). Each of the newly “born” individuals then has the option to convert to a new species with fixed speciation probability *ν* (boxes 4 and 5). If this occurs, a new species containing one individual is added to the population (box 6); otherwise the original species is updated to add another individual (box 7). The process is then repeated with the updated population vectors as input. To ensure convergence, for each model the simulations were run until the two starting population conditions had converged on the same species abundance distribution (allowing for stochastic variation).

The simulation model described above generates the same outcome as the algorithm implemented in the untb package [7, 30] (see previous section). It additionally provided a framework that can be altered to reproduce the mechanisms outlined in Hypotheses 1–4 (MVLA-type speciation/selection, unsampled reservoir population, increasing prevalence, and superspreading), and to assess to what extent they are able to replicate the manner in which the observed distribution of NI MVLA-types diverged from NTB predictions. The individual models themselves are described in the following section.

On completion of a simulation, the value of the biodiversity number *θ* was estimated for each model run using the untb package, and in order to summarise the runs for each model and to assess their divergence from NTB these values of *θ* were then averaged across all runs for each specific model. Predictions of the RAD expected under NTB were generated in the untb package as above using this average value of *θ*, and the 95th percentile interval of the NTB predictions was then plotted and compared to the 95th percentile interval of the simulation results.

The parameters for the baseline model were chosen to approximate the situation in NI, with each timestep approximately representing 1 week. Using data on the numbers of typed breakdowns over the study timeframe the number of deaths, *dJ*_*M,t*_, and the number of births, *bJ*_*M,t*_, per timestep were both set to 24 individuals (equivalent to the mean herd-level incidence of MVLA-typed breakdowns per week; Additional file 3 grey line); the population size *J*_*M,t*_ was set to 890 individuals (equivalent to the mean herd-level breakdown prevalence across NI; Additional file 3 black line); and the speciation probability *ν* as set as 0.015 (equivalent to the mean proportion of new MVLA-typed breakdowns attributable to novel MVLA-types; Additional file 4). Although the values of *d*, *b*, *J*_*M,t*_ *,* and *ν* were not formally fitted to the data, these parameters form an intrinsic part of NTB. Varying these should not alter the qualitative agreement between the simulation results and the NTB predictions, except where *b* is not equal to *d*: this latter scenario is explored in the Hypothesis 3 (increasing population size) simulations described below.

The model for Hypothesis 1 (MVLA-type speciation and/or choice of loci) assumes a stepwise mutation process, but none of the other models described below discriminate between new species occurring through mutation or through immigration from outside the simulated community. For all models with the exception of Hypothesis 4 (superspreading), the number of new births in each species is directly proportional to the frequency of the species in the population, after accounting for stochastic variation (box 4 Fig. 1).

Stochastic simulations representing each of the four hypotheses were run 500 times (250 runs starting with all individuals of the same species, and 250 runs starting with all individuals as different species), and the results were summarised to give output equivalent to the incidence of (MVLA-typed) breakdowns over the course of a year (timesteps corresponding to 52 weeks).

### Hypothesis 1 - MVLA-type speciation and/or choice of MVLA-loci

Mutation in MVLA-type occurs through variation in the number of repeats present at the MVLA loci. This process can give rise to homoplasies, with unrelated lineages converging on the same MVLA-type [23], and this could act to augment the more common MVLA-types. To test whether homoplasy in MVLA-types and/or the method by which MVLA-typing loci were chosen in NI would affect the RAD of different MVLA-types, the basic model above was altered so that species was denoted by a string of 12 integers. The 12 integers represent the number of tandem repeats present at the 12 candidate MVLA-loci from which the current NI typing loci were originally selected [14]. In simulations started with all individuals in the same species, all 12 “loci” were set to 5 for all individuals, while in simulations started with all individuals in different species, each of the 12 loci for each individual was chosen as a randomly sampled integer between 1 and 10. When a speciation event occurred in the model (box 6 Fig. 1), this generated a stepwise mutation in MVLA-type, with one “locus” chosen at random from the string of 12, and the number of repeats at this locus then increased or decreased by one. Note that in this simulation we do not address the scenario that different MVLA-loci may have differential rates of gain and/or loss of repeats [32].

Since we assume that each locus has an equivalent chance of gaining/losing a repeat and so generating a new species, having more MVLA loci will proportionally increase the overall rate of speciation. Therefore, as we consider 12 loci here (as opposed to the seven loci that determined the rate of occurrence of new species in Additional file 4 and the basic model), for these simulations, the speciation rate *ν* was proportionally increased to 0.0257 per birth event. All other parameters were as described above.

On completion of each simulation, to mimic the manner in which the MVLA-typing loci were chosen in NI [14], the seven most diverse “loci” from each model run were chosen using the Hunter-Gaston index, a measure of the probability that two individuals sampled at random are of the same type when assuming sampling without replacement [33]. This is a commonly used measure of diversity in microbiology, and was used in the selection of MVLA-typing loci in NI [14]. Following identification of the most diverse loci, the species designation of each individual in the model output was then re-named based solely on these seven loci and comparison to NTB predictions was carried out as above.

### Hypothesis 2 - Unsampled reservoir of infection

**Fig. 2:**
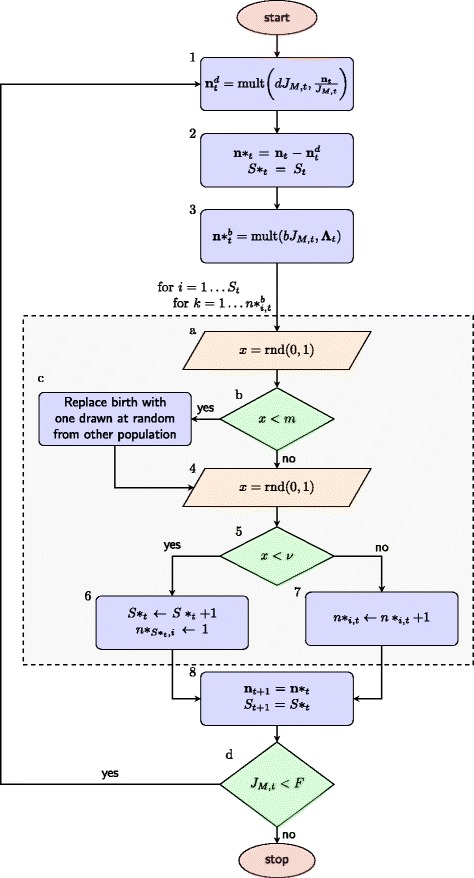
Flowchart based on Fig. 1 (basic model), showing the modifications made to simulated Hypotheses 2–4. Boxes 1–8 are similar to Fig. 2. For Hypothesis 2 (unsampled reservoir), parameter *m* (box b) represents the likelihood of an individual migrating into the system from the separately modelled population (box c). For hypothesis 3 (increasing population size), parameter *F* (box d) represents the final target population size for the model. For Hypothesis 4 (superspreading), ***Λ***_*t*_ (box 4) gives the vector of the probability of reproduction for each species, which is the sum of the reproduction probabilities *λ*, for each individual *j* in each species *I*, divided by the sum of reproduction probabilities for all individuals in the system

Hypothesis 2 posited that an unsampled reservoir of *M. bovis* infection might account for the observed RAD of MVLA-types in NI. To test this, we extended the baseline model to consider two separate populations of *M. bovis*, linked through immigration of individuals between them (Fig. 2, boxes b and c).

We consider this hypothesis in terms of an unsampled badger reservoir for illustrative purposes, however the same processes could also easily be represented by another type of unsampled population. The MVLA-type abundances reported here were sampled solely from the cattle population. Assuming bi-directional transmission between cattle and badgers (Additional file 5), in infected badgers, novel MVLA-types may emerge through speciation from types circulating in badgers or through import of strains from the cattle population, whereas in cattle novel MVLA-types could occur through speciation from circulating cattle strains, import from the badger population, and also additionally through import of MVLA-types circulating in cattle in other countries. Therefore the rate of occurrence of new MVLA-types may differ between cattle and badgers, and this difference in the occurrence of novel MVLA-types could affect the RAD observed.

In this model we make no distinction between novel species occurring as a result of speciation or immigration, both being combined within the speciation rate parameter *ν*. To mimic the effects of differing rates of the occurrence of novel MVLA-types between simulated badger and cattle infections, different values of speciation rates *ν* were investigated for the target population (representing infections in cattle) and the linked population (representing infection in badgers), and values of migration *m* between the two populations were also varied. Migration rates were tested for values of 0.0208 (representing approximately one migration per timestep) and 0.0833 (approximately two migrations per timestep). Increasing the rate of migration between the populations brings the simulation closer to an homogeneously mixing population, and therefore would be expected to result in RADs more similar to that predicted under NTB. Table 2 shows the combinations of these parameters examined here. Except for these parameters the two simulated populations were identical.

**Table 2:** Combinations of parameter values used in simulations for the Hypothesis 2 model (unobserved reservoir of infection)

	*ν*_*t*_	*ν*_*l*_	*m*_*l,t*_	*m*_*t,l*_
A	0.01	0.01	0.0208	0.0208
B	0.02	0.01	0.0208	0.0208
C	0.1	0.01	0.0208	0.0208
D	0.01	0.01	0.0208	0.0833
E	0.02	0.01	0.0208	0.0833
F	0.02	0.01	0.0833	0.0208

Speciation rates for the target population (*ν*_*t*_) and for the linked population (*ν*_*l*_) were varied as were migration rates from the target population to the linked population (*m*_*l,t*_) and from the linked population to target population (*m*_*t,l*_) . Six combinations of these values were tested, labelled A-F

### Hypothesis 3 – Recent expansion of the *M. bovis* population

To investigate the effects of historical bTB increases on the RAD of MVLA-types, the basic model was altered to approximate the increase in bTB prevalence that has affected NI in the decades prior to 2002 [26].

Based on data from [12], peak bTB levels in NI were estimated at approximately 3200 herd breakdowns in 2002. To keep the results of this model proportional to our other simulations we took 57.5% of this value (the proportion of NI herd breakdowns linked to at least one molecular typing record in the 2003–2010 data), giving 1840 breakdowns as the final (maximum) population size for the increasing population size model. Data available from http://www.bovinetb.info/docs/number-of-bovine-tb-reactors-slaughtered-in-northern-ireland-from-1961-to-nov-2011.pdf (originally made available by the Department of Agriculture and Rural Development NI) provide historic summaries of numbers of cattle testing positive to bTB (Additional file 6). These figures do not include bTB infections identified at slaughter, however we took them to broadly represent historical trends, which would indicate that the increase in bTB in NI started in 1986, at which point bTB levels were approximately 9.3% of their maximum.

The basic constant-size simulation model described above was first run at the starting population size *J*_*M*,0_ = 171 for 100,000 timesteps to allow equilibration of the simulated RAD. A model simulating increasing population size was then run starting from this population, until the target end population size (1840*,* box d *F*, Fig. 2) was reached. To generate an exponentially increasing population size approximately equivalent to the historical situation in NI, the model was run with death rate *d* = 0.02697 (box 1 Fig. 2) and birth rate *b* = 0.02883 (box 3, Fig. 2). Varying the end population size (*F)* would alter the rate of population increase over the simulation: a higher value of *F* would lead to a higher rate of population increase, which would be expected to exacerbate any divergences between the simulation results and those predicted under NTB prediction.

### Hypothesis 4 - Superspreading

To assess the effect of superspreading on the RAD of NI MVLA-types, the basic model was extended to incorporate transmission heterogeneity through the introduction of systematic variation in the number of offspring per individual breakdown. Superspreading in this context is taken to represent variability in the number of herds infected by each infected herd, however we do not distinguish between the various possible causes of this herd-level transmission heterogeneity.

To simulate this transmission heterogeneity, at the point at which new births were identified in the model (box 3 Fig. 2), each new birth was also assigned an individual reproduction number, *λ*, which was then retained throughout the lifespan of that individual. This individual reproduction number was drawn from a gamma distribution with a mean of 1 (equal to the effective reproduction rate in a population with a constant prevalence) and with shape parameter *k*, after [34]. Simulations were run for *k* values of 0.1, 0.5, 1, and 10, to assess the effect of different degrees n transmission heterogeneity on model outputs. As before, a set number of individuals died (*dN*_*t*_ = 24) and were born (*bN*_*t*_ = 24) at each timestep, however, in this model the chance of an individual being chosen to reproduce at each timestep was directly proportional to its pre-assigned reproduction probability *λ*, and therefore Λ_*i,t*_, the number of individuals of species *i* reproducing at each timestep *t*, is determined by the sum of the reproduction numbers *λ* for each individual *j* in species *i* divided by the sum of the reproduction probabilities for all individuals in the population:

$$ {\varLambda}_{i,t}=\frac{\sum_{j=1}^{N_{i,t}}{\lambda}_{i,j}}{\sum_{i=1}^{n_t}\sum_{j=1}^{N_{i,t}}{\lambda}_{i,j}} $$

The number of offspring generated by a single individual over the course of its lifespan is related to the reproduction number of the individual as well as the lifespan of the individual (as death rate *d* is constant, the individuals’ lifespan will follow a geometric distribution).

## Results and discussion

### Null hypothesis - neutral ecological drift

**Fig. 3:**
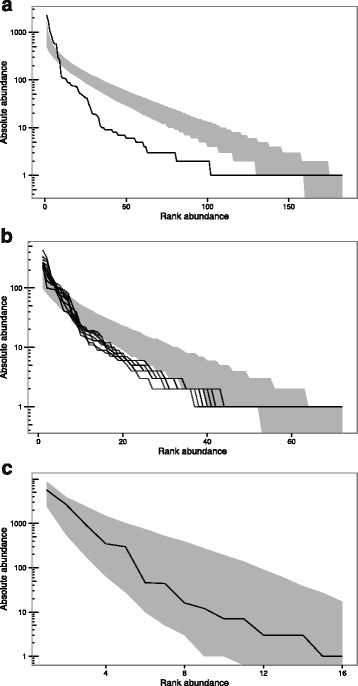
Comparison of observed relative abundance distributions (lines) and 95% envelopes for neutral predictions (shading) for observed distributions of molecular types for: NI *M. bovis* MVLA-types aggregated across the study period (**a**); NI *M. bovis* MVLA-types separated by year (**b**); NI *M. bovis* spoligotypes aggregated across the study period (**c**). Graphs show log-scaled absolute abundance of each molecular type on the y-axis, compared to the ranked abundance of each type on the x-axis (where the most common types have a rank of 1, and increasing ranks indicate less abundant types)

The null model of neutral ecological drift described by NTB [7, 8] was unable to explain the observed relative abundances of *M. bovis* MVLA-types recorded in NI, whether considering the distribution across the whole study period (Fig. 3a), or when divided into separate years (Fig. 3b). Although previous work has shown that individual MVLA-types in NI have expanded or contracted significantly over time [13], we found no obvious differences in the MVLA-type RADs observed over different years of the study period (Fig. 3b). In the observed MVLA-type distributions, the common types appear more dominant than would be expected under NTB, with a tail of MVLA-types that are more rare than expected. The RAD of *M. bovis* spoligotypes in NI does not fall outside the 95th percentile interval of the NTB predictions (Fig. 3c), however, with lower numbers of spoligotypes than MVLA-types the predicted neutral distributions show greater variation and therefore the power to detect deviations from neutral expectations is reduced for the spoligotype RAD.

### Hypothesis 1 - MVLA-type speciation and/or selection of MVLA-loci

**Fig. 4:**
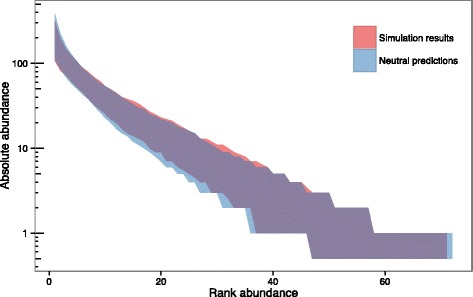
Comparison between Hypothesis 1 simulations (MVLA-type speciation and choice) and neutral predictions. The 95% envelope for the relative abundance distributions generated by 500 simulations is shown in red, and the 95% envelope for predictions under neutral theory in blue. Log-scaled absolute abundance is shown on the y-axis and ranked abundance on the x-axis (species ranked in order of decreasing abundance)

The process through which MVLA-types are identified and new types arise is based on variation in the number of repeats present at different MVLA loci. This gives rise to the possibility of unrelated lineages converging on the same MVLA-type [23], which could act to augment the more common MVLA-types. Additionally, the MVLA-loci used for MVLA-typing in NI were originally chosen from a panel of 12 candidate loci to optimally discriminate the *M. bovis* population in NI [14]. It was unclear whether either or both of these processes would affect the RAD of MVLA-types and their fit to neutral predictions. The results of the simulations replicating these processes show minimal differences from the distribution predicted under NTB (Fig. 4), and therefore it appears that the process of MVLA-type speciation and the manner in which the panel of MVLA-typing loci were chosen in NI cannot account for the shape of the observed RAD seen in NI MVLA-types.

### Hypothesis 2 - Unsampled reservoir of infection

**Fig. 5:**
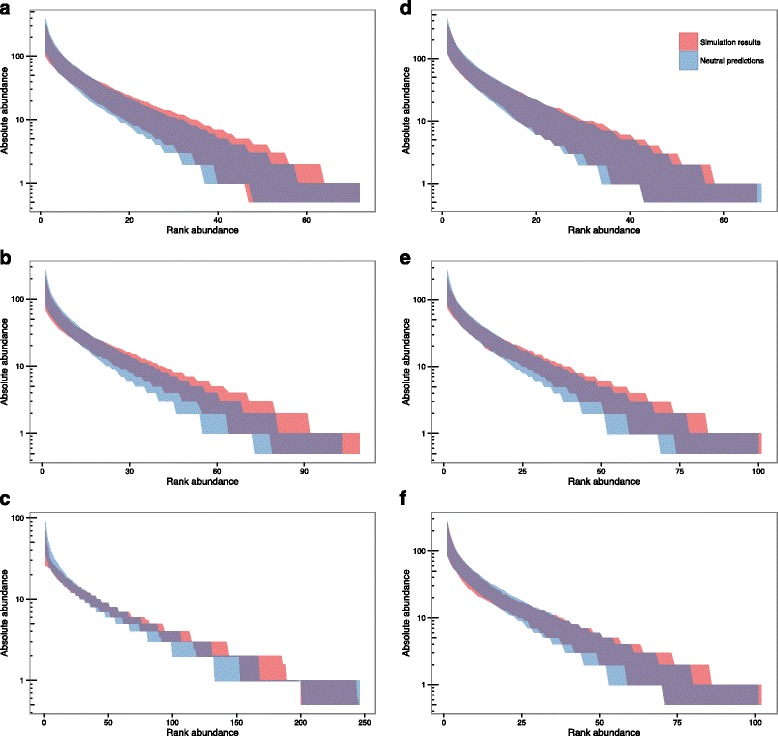
Comparison between Hypothesis 2 simulations (unobserved reservoir) and neutral predictions, plotted for the target (cattle) population. Results are shown for: a model with speciation probability per birth in the target (cattle) population, *ν*_*t*_, of 0.01 and speciation probability per birth in the linked (badger) population, *ν*_*l*_, of 0.01. Migration probabilities (*m*_*l,t*_ and *m*_*t,l*_) were 0.0208 (approx. One migration every two timesteps) in both directions (**a**); a model with *ν*_*t*_ = 0.02; *ν*_*l*_ = 0.01; *m*_*l,t*_ = 0.0208; *m*_*t,l*_ = 0.0208 (**b**); a model with *ν*_*t*_ = 0.1; *ν*_*l*_ = 0.01; and *m*_*l,t*_ = *m*_*t,l*_ = 0.0208 (**c**); a model with *ν*_*t*_ = *ν*_*l*_ = 0.01; and *m*_*l,t*_ = 0.0208; and *m*_*t,l*_ = 0.0833 (approx. Two migrations per timestep) (**d**); a model with *ν*_*t*_ = 0.02; *ν*_*l*_ = 0.01; *m*_*l,t*_ = 0.0208; and *m*_*t,l*_ = 0.0833 (**e**); and for model with *ν*_*t*_ = 0.02; *ν*_*l*_ = 0.01; *m*_*l,t*_ = 0.0833; and *m*_*t,l*_ = 0.0208 (**f**). The 95% envelope for the relative abundance distributions generated by 500 simulations is shown in red, and the 95% envelope for predictions under neutral theory in blue. Log-scaled absolute abundance is shown on the y-axis and ranked abundance on the y-axis (species ranked in order of decreasing abundance)

All molecular typing results described in this study originated from cattle infections in NI, where only limited information is available on *M. bovis* types infecting hosts other than cattle, such as badgers. The results of the model simulating an unsampled reservoir suggested that this scenario in itself could not account for the observed distribution of *M. bovis* MVLA-types in NI, a result which held true even when the rate of occurrence of novel MVLA-types was allowed to differ between the two host populations (Fig. 5). In fact, this simple metacommunity structure actually generated RADs that were slightly more even than that predicted by NTB, for all combinations of migration and speciation probabilities explored here.

Similar findings have been reported for spatial structuring of a community via spatially limited dispersal of individuals [4], which likewise generates more even distributions of species across the metacommunity than predicted by NTB in the absence of dispersal limitation. We therefore suggest that a reservoir of infection or other forms of metacommunity structure, including the spatial structuring of the *M. bovis* population, would (in the absence of other factors) be expected to draw the RAD in the opposite direction to that seen in the NI MVLA-type data, acting to even out the skewed distribution observed.

The absence of a more even distribution of MVLA-types in the observed data does not rule out metapopulation structure (either as a reservoir of infection in a different species, and/or through other forms of population structuring) in the epidemiology of bTB in NI. In fact, many lines of evidence point towards a reservoir of bTB infection in the badger population [35], and spatial structuring of the *M. bovis* population is evident in the geographical clustering of molecular types recorded in NI [13]. Rather, the observed pattern of the MVLA-type RADs indicates that, if some form of population structure is involved in the spread of *M. bovis*, other processes must also be acting to overcome its levelling influence and push the RADs towards the skewed distributions present in these data (Fig. 3).

### Hypothesis 3 - recent expansion of the *M. bovis* population

The available data on numbers of cattle testing positive for bTB in NI suggest the prevalence of bTB increased from the late 1980s to 2002 (Additional file 6, also [26]). One of the key assumptions made by NTB is a constant size population [7], and Luciani et al. demonstrated that violating this assumption through an increasing population size (i.e. increasing prevalence) could generate the patterns of relative abundances recorded in an outbreak of human TB in California [9].

**Fig. 6:**
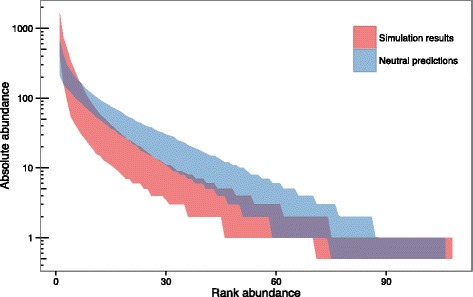
Comparison between Hypothesis 3 simulations (increasing population size) and neutral predictions. The 95% envelope for the relative abundance distributions generated by 500 simulations is shown in red, and the 95% envelope for predictions under neutral theory in blue. Log-scaled absolute abundance is shown on the y-axis and ranked abundance on the x-axis (species ranked in order of decreasing abundance)

The results of the Hypothesis 3 simulations (Fig. 6) agree with this, demonstrating that an increase in population size, approximating historical increases in bTB prevalence in NI, is capable of generating a RAD that deviates from NTB predictions in a similar manner to that of observed distribution of NI MVLA-types. However, since 2002 levels of bTB have been declining in NI (Additional file 6, [26]). Model simulations mimicking a declining population (without the prior increase) indicated that a decreasing prevalence would have the opposite effect, generating more even RADs than expected under NTB (results not shown). Thus the more recent decrease in bTB prevalence is likely to even out to some extent the skew in RAD generated by earlier increases.

### Hypothesis 4 – Superspreading

Variation in the number of secondary infections generated by infected individuals, or superspreading, is manifest in the epidemiology of a range of diseases [34, 36]. Superspreading has also been implicated in the spread of bTB in Britain, with a minority of infected cattle herds thought to be responsible for a large proportion of the onward transmission of the disease [27]. Ypma et al. also demonstrate that superspreading could account for skewed abundances of molecular types of human TB in the Netherlands [10].

**Fig. 7:**
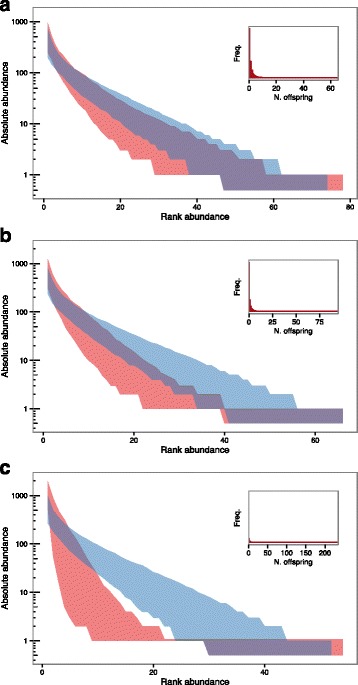
Comparison between Hypothesis 4 simulations (superspreading) and neutral predictions for *k* values of 1, 0.5, and 0.1 (**a**, **b**, and **c** respectively). The 95% envelope for the relative abundance distributions generated by 500 simulations is shown in red, and the 95% envelope for predictions under neutral theory in blue. Log-scaled absolute abundance is shown on the y-axis and ranked abundance on the x-axis (species ranked in order of decreasing abundance). Inset graphs show the number of individuals that give rise to different numbers of offspring, averaged over all simulations, for each value of *k*

The results of superspreading simulations where individuals’ chances of reproduction were distributed with mean of 1 and *k* values of 1, 0.5, and 0.1 are shown in Fig. 7a–c, respectively, with the distribution of the number of offspring per individual over the course of the simulations given in inset graphs. These results indicate that superspreading at these levels could also generate divergences from NTB similar to that seen in the RAD of MVLA-types in NI (Fig. 3a and b).

## Conclusions

In this study we show that a simple model of neutral ecological drift cannot account for the observed distribution of NI MVLA-types, and that the observed data showed a more skewed distribution than expected under NTB. Simulation models were then used to identify which, if any, of four specific biologically-plausible hypotheses could reproduce the manner in which the observed RAD diverged from NTB predictions. We note that the hypotheses examined in this study are not mutually exclusive, and were specifically chosen due to the likelihood that they are all involved in the epidemiology and evolution of *M. bovis* in NI.

We conclude that MVLA-type homoplasy and the manner in which MVLA-typing loci were selected in NI, taken in isolation, have little impact on the expected abundances of different MVLA-types. By contrast, structuring of the community, for example due to a reservoir population or alternatively through processes such as spatially limited dispersal of individuals [7], is likely to generate a more even RAD than expected under NTB. The finding of a very uneven distribution of MVLA-types despite the probable involvement of the latter scenarios in bTB epidemiology in NI indicates that processes other than these must be involved and responsible for generating the skewed RAD recorded in these data. The results presented here for Hypotheses 3 and 4 indicate that some combination of historical increases in the prevalence of bTB in NI and/or variation in the number of onward transmission events generated by each infected herd (i.e. superspreading) may have contributed to the unevenness evident in the distribution of *M. bovis* MVLA-types in NI. Studies of the RAD of human TB also concluded that an increasing population size [9] and superspreading [10] may be responsible for the skewed distribution of molecular types observed in some human TB datasets.

None of the various hypotheses tested here break the basic tenet of neutral drift, as in each simulation different molecular types were considered equal and interchangeable, although they did deviate from the original assumptions of Hubbell’s NTB in other ways. Another potential mechanism through which the observed skew in distribution of MVLA-types could be generated is through selective pressures acting on different molecular types, with more fit types present at higher than expected abundance and less fit types rarer than expected. This has been explored extensively in the ecological literature as the main alternative hypothesis to neutral theory [8], and was also put forward by Smith et al. [15] as a possible explanation for the distribution of molecular types of *M. bovis* in GB. Although not modelled here, this remains an additional explanation for the observed pattern present in the MVLA-type data. However, in a low diversity population such as that of *M. bovis* in NI, selective pressures are not expected to play a strong role in driving the patterns of diversity observed, and our results demonstrate that other processes as outlined above can generate similar skewed distributions without needing to invoke selective pressures.

Data on the abundances of different molecular types are relatively easy to obtain for many pathogens of veterinary and human importance. We demonstrate how the rich body of work in ecology relating to relative abundances of different species in an ecosystem can be used to test hypotheses regarding the driving forces underlying the distribution of relative abundances of different pathogen molecular types. Although RADs in isolation do not definitively disentangle the relative roles of the processes under consideration, they do enable the identification of candidate mechanisms likely to be involved in driving the observed patterns, as we show here. Combining these approaches with additional data on, for example, spatial and genetic relationships between samples may allow more detailed discrimination between candidate processes, however other methodologies may be more appropriate to fully understand the relative roles of the different processes involved in the system.

## Additional files

Additional file 1: Table of number of breakdowns recorded for each NI spoligotype, 2003–2010 (XLS 65 kb)

Additional file 2: Table of number of breakdowns recorded for each NI MVLA-type, 2003–2010 (XLS 65 kb)

Additional file 3: Graph showing prevalence (black) and monthly incidence (grey) of MVLA-typed herd breakdowns in NI over the study period. (PDF 19 kb)

Additional file 4: Graph showing number of breakdowns attributed to novel MVLA-types per month in NI over the study period (PDF 14 kb)

Additional file 5; Summary of the linked cattle-badger population and the source of new MVLA-types in each. (PDF 217 kb)

Additional file 6: Graph of historical incidence of cattle testing positive for bTB in NI. (PNG 55 kb)
