# Supplementary figures and images for: Relative abundance of Mycobacterium bovis molecular types in cattle: a simulation study of potential epidemiological drivers
Source: BMC Vet Res. 2017 Aug 22;13:268. doi: 10.1186/s12917-017-1190-5 (PMC5567634; doi:10.1186/s12917-017-1190-5)

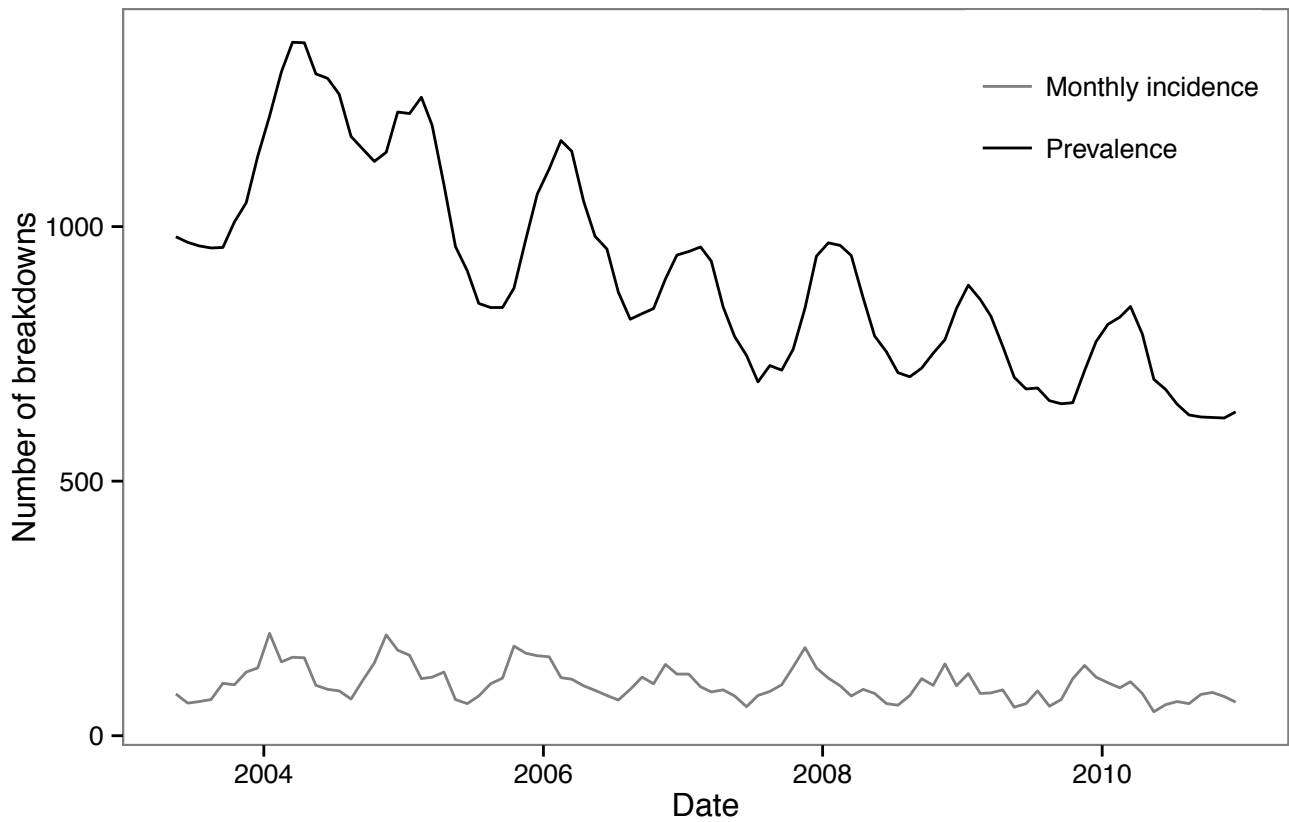

Supplement: Supplementary file 3 — Graph showing prevalence (black) and monthly incidence (grey) of MVLA-typed herd breakdowns in NI over the study period. (PDF 19 kb) [file 12917_2017_1190_MOESM3_ESM.pdf]

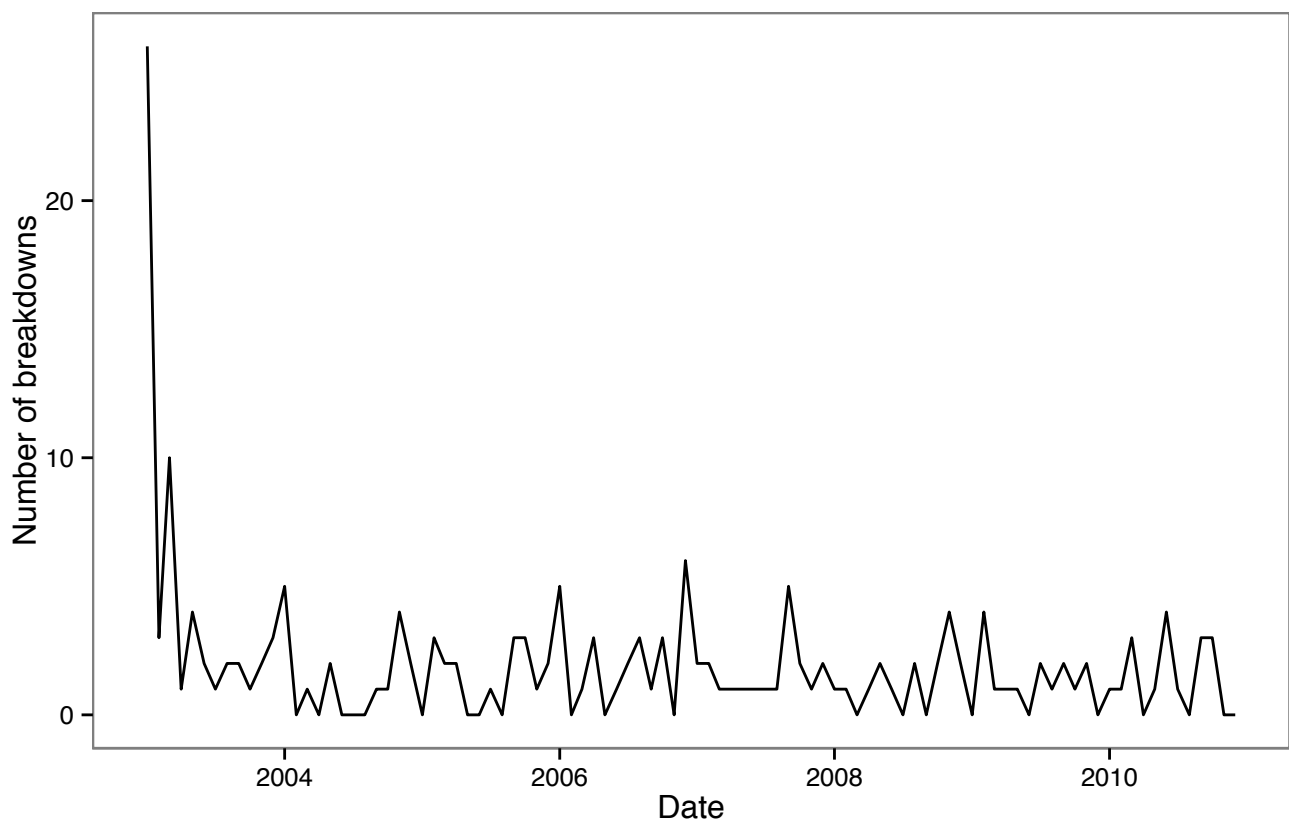

Supplement: Supplementary file 4 — Graph showing number of breakdowns attributed to novel MVLA-types per month in NI over the study period (PDF 14 kb) [file 12917_2017_1190_MOESM4_ESM.pdf]

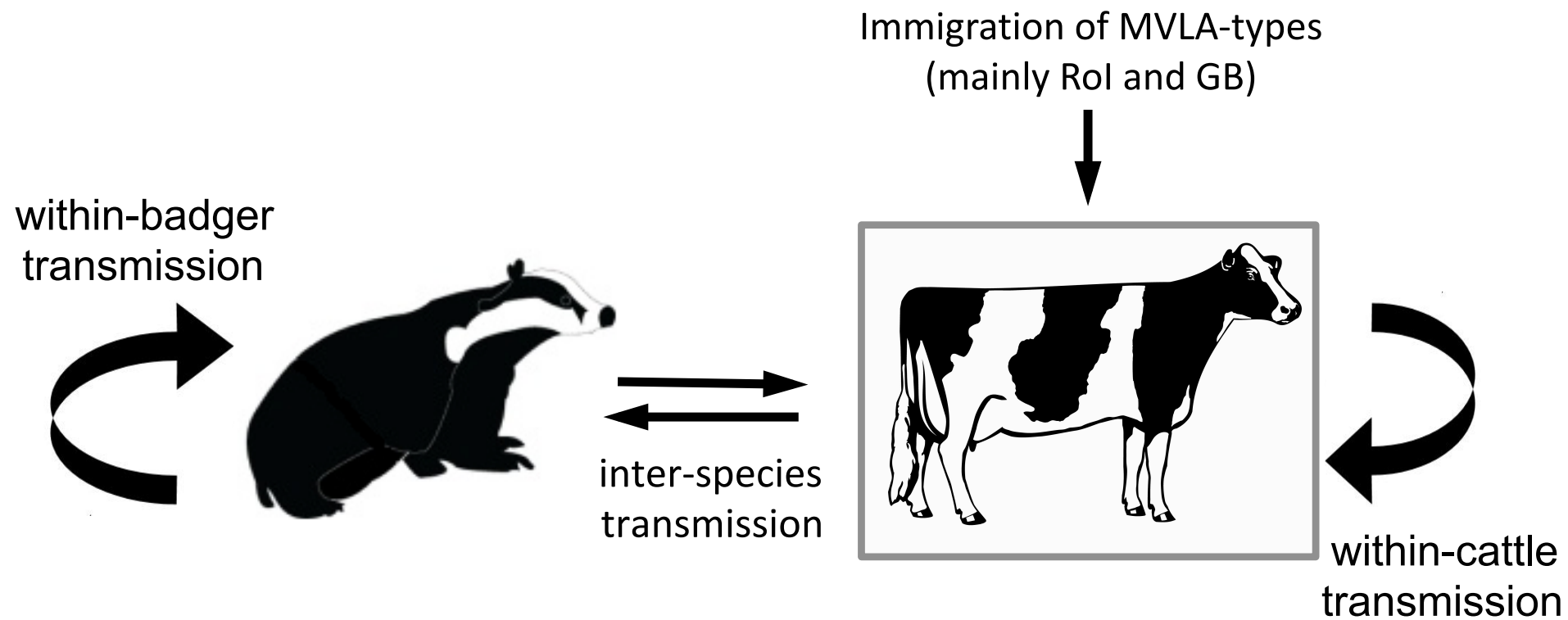

Supplement: Supplementary file 5 — Summary of the linked cattle-badger population and the source of new MVLA-types in each. (PDF 217 kb) [file 12917_2017_1190_MOESM5_ESM.pdf]

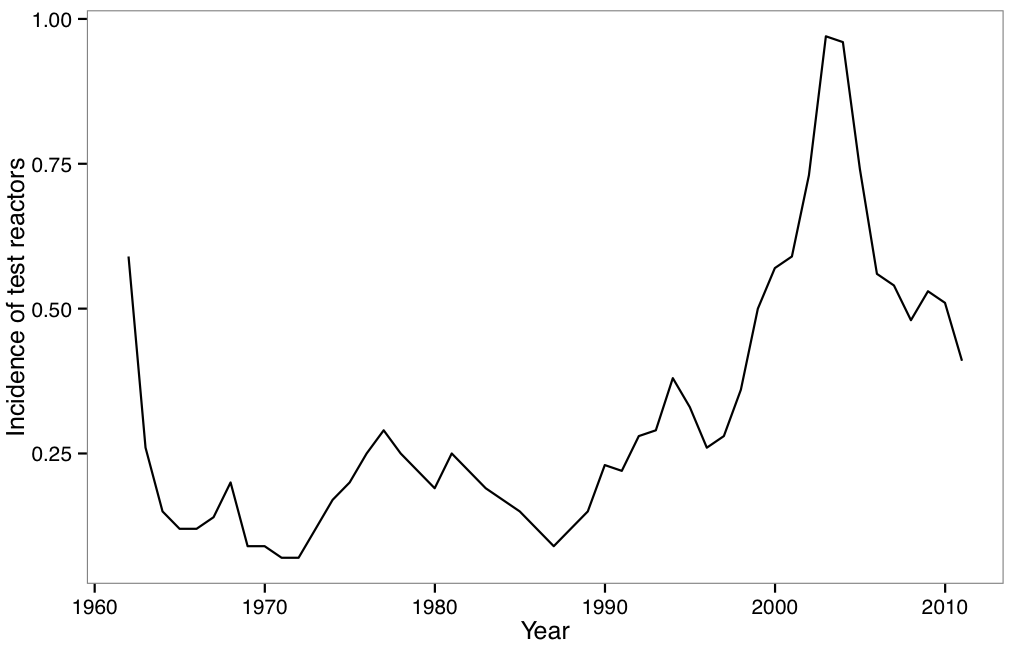

Supplement: Supplementary file 6 — Graph of historical incidence of cattle testing positive for bTB in NI. (PNG 55 kb) [file 12917_2017_1190_MOESM6_ESM.png]
